# Identification of novel resistant sources for ascochyta blight (*Ascochyta rabiei*) in chickpea

**DOI:** 10.1371/journal.pone.0240589

**Published:** 2020-10-19

**Authors:** Gayacharan, Upasana Rani, Sarvjeet Singh, Ashwani K. Basandrai, Virender K. Rathee, Kuldeep Tripathi, Neeta Singh, Girish P. Dixit, Jai C. Rana, Sushil Pandey, Ashok Kumar, Kuldeep Singh

**Affiliations:** 1 ICAR-National Bureau of Plant Genetic Resources, New Delhi, India; 2 Department of Plant Breeding and Genetics, Punjab Agricultural University, Ludhiana, Punjab, India; 3 Rice and Wheat Research Centre, CSKHPKV, Malan, Himachal Pradesh, India; 4 Hill Agricultural Research and Extension Centre, CSKHPKV, Dhaulakuan, Himachal Pradesh, India; 5 ICAR-Indian Institute of Pulses Research, Kanpur, India; 6 Bioversity International, India Office, NASC, New Delhi, India; National Institute for Plant Genome Research, INDIA

## Abstract

Chickpea (*Cicer arietinum* L.) is the second largest pulse crop grown worldwide and ascochyta blight caused by *Ascochyta rabiei* (Pass.) Labr. is the most devastating disease of the crop in all chickpea growing areas across the continents. The pathogen *A*. *rabiei* is highly variable. The resistant sources available are not sufficient and new sources needs to be identified from time to time as resistance breakdown in existing chickpea varieties is very frequent due to fast evolution of new pathotypes of the pathogen. Therefore, this work was undertaken to evaluate the existing chickpea germplasm diversity conserved in Indian National Genebank against the disease under artificial epiphytotic conditions. An artificial standard inoculation procedure was followed for uniform spread of the pathogen. During the last five *winter* seasons from 2014–15 to 2018–19, a total of 1,970 accessions have been screened against the disease and promising accessions were identified and validated. Screening has resulted in identification of some promising chickpea accessions such as IC275447, IC117744, EC267301, IC248147 and EC220109 which have shown the disease resistance (disease severity score ≤3) in multiple seasons and locations. Promising accessions can serve as the potential donors in chickpea improvement programs. The frequency of resistant and moderately resistant type accessions was comparatively higher in accessions originated from Southwest Asian countries particularly Iran and Syria than the accessions originated from Indian sub-continent. Further large scale screening of chickpea germplasm originated from Southwest Asia may result in identifying new resistant sources for the disease.

## Introduction

Chickpea (*Cicer arietinum* L.), is a self-pollinated, diploid (2n = 2X = 16) annual legume which ranks second worldwide after soybean as a food legume crop [1]. It is one of the oldest crops cultivated by man. Archaeological evidences of chickpea dates back to 7,500–6,800 BC were found in the Middle East. More precisely south-eastern Turkey and adjoining Syria are considered as the centre of origin of the crop [2, 3]. Chickpea is cultivated mainly in arid and semi-arid areas of more than 50 countries across Asia, Africa, Europe, Australia, North America and South America [4]. Globally 17.2 million tonnes of chickpea is produced from ca. 17.8 m ha land which is ca. 15% of total pulse area well as production. The crop is reported to be susceptible to more than a dozen of well documented pathogens [5]. Among them Ascochyta blight (*Ascochyta rabiei* Pass. syn. *Phoma rabiei* Pass., *Didymella rabiei* Kovatsch.) is the most devastating disease [6]. The pathogen can also infect wild *Cicer* species like *Cicer montbretti*, *Cicer ervoides*, *Cicer judaicum*, *Cicer pinnatifidum*, etc [7]. The disease was first observed during 1911 in the North-West Frontier Province region of India, which is now part of Pakistan [8]. Since then the pathogen has spread in almost all chickpea growing regions in the world. The disease has been reported from 34 countries across the six continents and is a major disease of west Asia, northern Africa and southern Europe [5, 9–11]. As the pathogen is the seed-borne in nature, the disease might have spread from its origin site to distant continents through chickpea germplasm exchanges. Stem breakage along with girdling and collapse of twigs and pod infection are the two most damaging symptoms of this disease. Pathogen’s spores spread through water splashes and wind [12]. Disease is more severe in areas where cool temperature and humid conditions prevail during the chickpea growing season.

Serious outbreaks of the disease had been witnessed from 1981 to 1983 resulted in wiping out of chickpea in northern parts of the country. As a consequence of the frequent epidemics, several prevalent landraces are threatened from the cultivation. During 1920–30 on an average 50% of the crop area in Attock district (now in Pakistan) and adjoining areas failed due to severe outbreak of this disease [13]. In Pakistan, the blight caused losses of nearly 50% of chickpea productions in consecutive three season from 1979–80 to 1981–82 [14]. Despite heavy application of pesticide (azoxystrobin), ascochyta blight caused ca. 20% yield losses in Nebraska, USA in 2001 on almost all the chickpea planted area [15]. Ascochyta blight was found to be the most devastating disease in chickpea growing regions of North China [16] and Ethiopia [17]. As the pathogen is seed borne, the disease has reached to non-traditional chickpea growing counties like Australia, Canada and other parts of the world and has become the major yield limiting factor [10]. This indicates the global importance of the disease. Recently several varieties have been bred with much better resistance to ascochyta blight, but still they require fungicidal application at flowering and pod formation stages [5, 18]. Moreover, the *A*. *rabiei* keep evolving and so it breaks down the host resistance systems in newly bred chickpea varieties [5, 19–21]. Therefore, new sources of resistance are required to sustain chickpea cultivation and production. Owing to the economic importance of the disease, the present study was aimed to evaluate the chickpea germplasm against this disease to identify novel sources and understand the level of ascochyta blight resistance available in chickpea collections.

## Materials and methods

### Source of germplasm and selection of experimental sites

**Fig 1:**
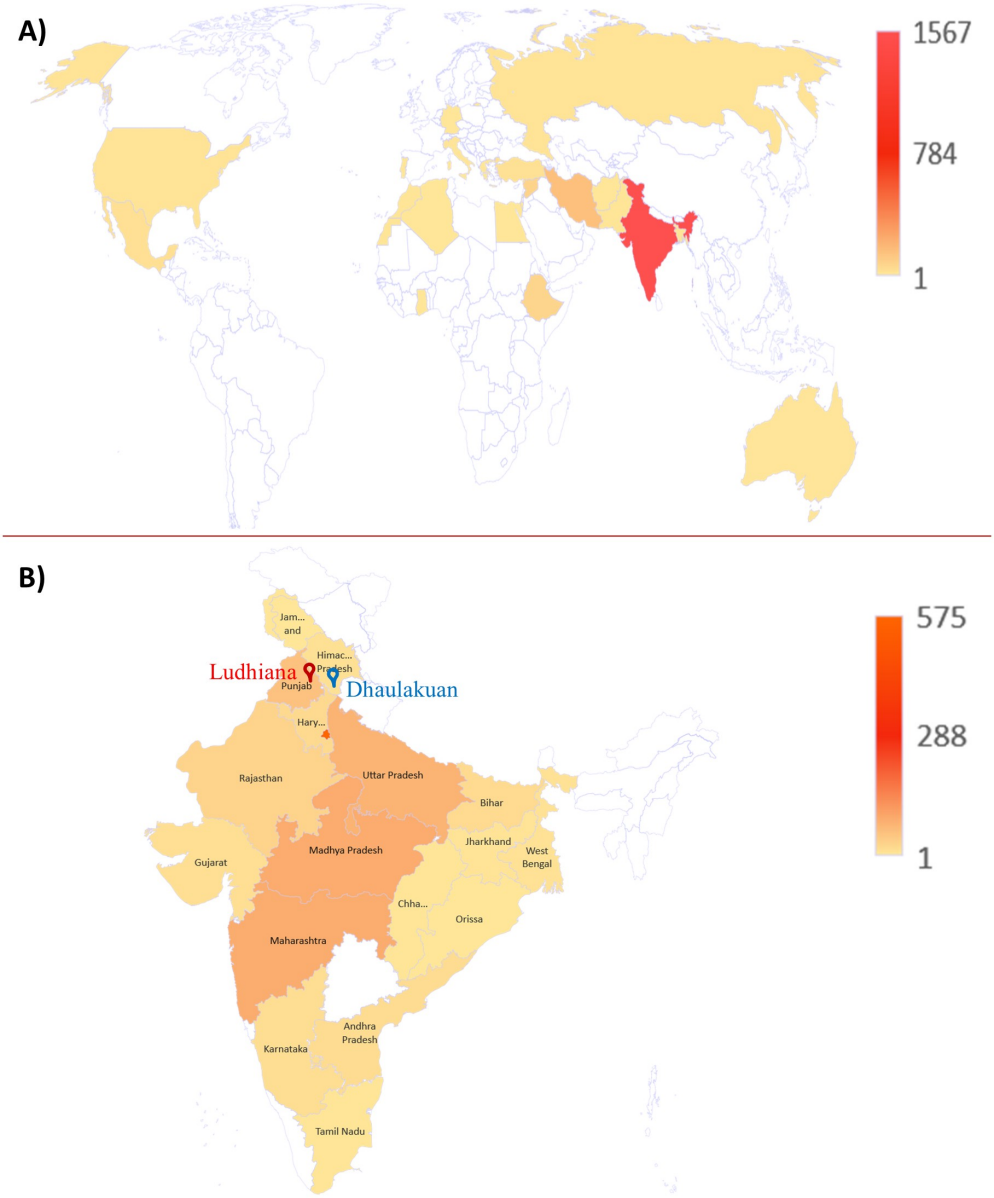
Representation of chickpea germplasm from 17 countries in world map (A) and 18 states (B) in Indian map. Colour intensity represents the number of accessions from the geographical location. Blue and red highlighted marks are the experimental locations in India.

**Fig 2:**
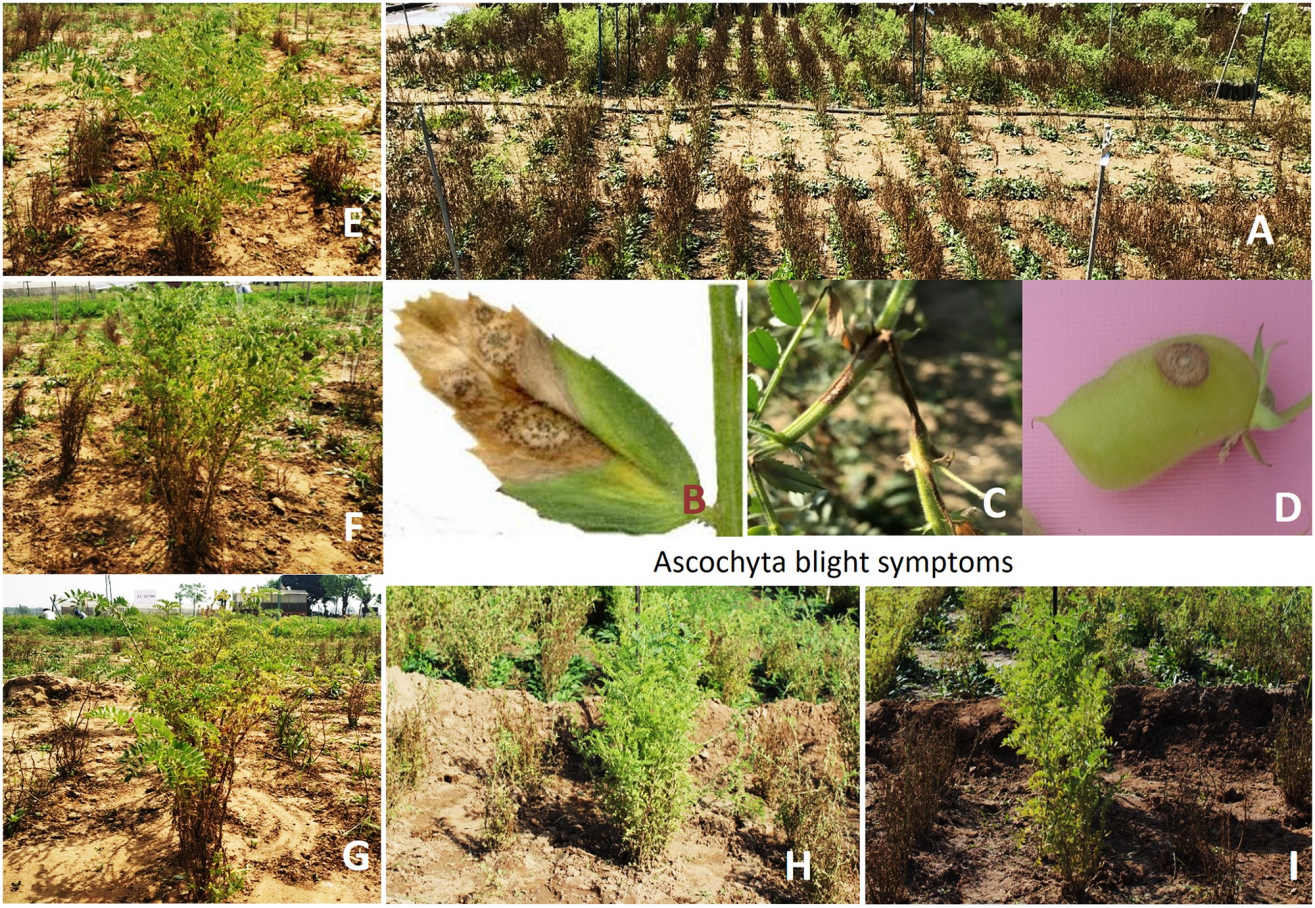
Field view of experimental plot and promising accessions. (A) Partial view of experimental plot depicting the disease severity and uniform spread of the pathogen (*A*. *rabiei*) at PAU, Ludhiana, during winter, 2018–19. Typical ascochyta blight disease identification symptoms which appear on leaf (B), stem (C) and pod (D) are highlighted in the photographs. Field photographs of some of the promising accessions *viz*. IC275447 (E), EC267301 (F), IC220109 (G), IC248147 (H) and IC117744 (I).

The chickpea germplasm lines were randomly selected and obtained from the National Genebank, ICAR-NBPGR, New Delhi. Accessions used in this study originated from 17 countries. Maximum number of the chickpea germplasm screened are of Indian origin (1,567 acc.) followed by Iran (155 acc.), Syria (75 acc.), Ethiopia (71 acc.), Mexico (26 acc.), Turkey (21 acc.) and other countries (55 acc.) (Table 1 & Fig 1). The detailed accession wise passport information is given in the S1 Data. The experiments were conducted at two locations i.e. Punjab Agricultural University, Ludhiana, India (30° 54' N and 75° 48' E) and HAREC, CSKHPKV, Dhaulakuan, India (30° 30' N and 77° 28' E). Both the locations are recognised as natural endemic regions for the *Ascochyta rabiei* due to optimal weather conditions for the disease spread during the season. Fig 2 highlights the disease severity and the uniformity of the pathogen spread throughout the experimental field. The same set of accessions were sown in both the locations in the winter seasons 2014–15, 2015–16 and 2016–17 and for last three seasons i.e. 2017–18, 2018–19 and 2019–20 only PAU, Ludhiana was the screening location. The detailed disease scoring data across the locations and seasons is given in S2 Data. And in each season new set of accessions were screened including the promising accession identified from earlier screening for validation. A total of 736, 250 and 250 accessions respectively were screened in both the experimental locations during winter season (November to March) of 2014–15, 2015–16 and 2016–17, while during 2017–18, 2018–19 and 2019–20, total 250, 325 and 308 accessions respectively were screened at PAU, Ludhiana. Over the years, 118 accessions were repeated to validate their consistency for disease severity over the years and locations. Some of the important validated accessions and their performance are listed in the Table 2. Screening was done utilizing Augmented Block Design (ABD) in which susceptible checks *viz*. L550 and JG62 were repeated after each fifth row alternately. Resistant check used was PB 5. These are commonly used chickpea varieties used as checks for ascochyta screening [22, 23]. Sowing was done on appropriate time in each winter season using recommended agronomic practices.

**Table 1:** Classification of chickpea germplasm based on their origin and disease severity frequency.

Country of origin	No. of accessions	R (1.1–3)	MR (3.1–5)	S (5.1–7)	HS (7.1–9)
Afghanistan	1				100.0
Algeria	1				100.0
Australia	3				100.0
Bangladesh	6				100.0
Egypt	1				100.0
Ethiopia	71			4.2	95.8
Germany	1				100.0
Ghana	1			100.0	
Greece	1				100.0
Iran	155	1.3	9.0	18.7	71.0
Israel	2			50.0	50.0
Italy	3			33.3	66.7
Mexico	26				100.0
Morocco	4				100.0
Pakistan	6			16.7	83.3
Portugal	1				100.0
Russia	8			12.5	87.5
Syria	75	4.0	10.7	12.0	73.3
Turkey	21			9.5	90.5
USA	8				100
India	1567	0.3	0.6	10.3	88.9
Unknown Exotic	8				100
**Total**	**1970**	**0.5**	**1.6**	**10.6**	**87.4**

**Table 2:** Disease reaction (0–9 score) to *Ascochyta rabiei* pathogen and basic passport information of promising chickpea germplasm accessions identified from screening of 1,705 chickpea germplasm from 2014–15 to 2018–19.

Genebank I.D.	Alternate identifier	Grain type	Date of collection	Origin	Ludhiana						Dhaulakuan			Disease severity (Mean)	Disease reaction
					2014–15	2015–16	2016–17	2017–18	2018–19	2019–20	2014–15	2015–16	2016–17		
IC117744	H-83-18	Desi	10/10/1991	India	-	2	-	2	2		-	1	-	1.75	R
EC267301	FLIP-8532	Kabuli	22/10/1988	Syria	-	-	3	-	3	3	-	1	-	2.5	R
IC275447	GL92057	Desi	31/05/1995	India	-	3	-	2	2	3	-	2	3	2.4	R
EC267309	FLIP84-35	Desi	22/10/1988	Syria	-	-	-	-	3	-	-	2	-	2.5	R
EC220109	ICC12023	Desi	31/10/1987	Syria	3	-	-	-	3	-	3	-	-	3	R
IC244185	BG323	Desi		India	-	5	-	-	-	-	-	2	2	3	R
IC209670	JG315	Desi	30/11/1997	India	-	-	5	-	-	-	-	-	1	3	R
IC248147	ICC4631	Desi	9/1/1973	India	-	-	-	2.5	3	3	5	-	-	3.37	MR
EC267186	BG-323	Desi	19/10/1988	Syria	-	-	5	-	3	5	-	2	-	3.75	MR
IC486423	ICC1058	Desi	9/1/1973	Iran	-	-	-	5	-	-	-	2	-	3.5	MR
EC223490		Desi	13/11/1987	Syria	-	5	5	-	5	-	-	4	1	4	MR
EC267240	ICC11871	Desi	22/10/1988	Syria	5	-	-	-	5	-	2	-	-	4	MR
EC223497	ICC4181	Desi	5/2/1980	Morocco	-	-	5	-	5	6	-	2	-	4.5	MR
IC209317	ICC3607	Desi	9/1/1973	Iran	-	5	4	6.5	5	7	-	4	4	5.0	MR
IC244433	ICCV93514	Kabuli		India	-	6	-	-	-	-	-	4	-	5	MR
ICC2792	P 29891	Desi	9/1/1973	Iran	-	-	5	5.5	7	-	-	-	4	5.37	S
IC486468	ICC1124	Desi	9/1/1973	Israel	-	-	-	8	-	-	3	-	-	5.5	S
IC373447		Desi	19/10/2002	India	-	-	6	6	-	-	-	-	5	5.67	S
IC552181	ICC12549	Desi	8/4/1983	Ethiopia	-	7	9	-	-	-	-	2	6	6	S
IC244505	NC-61179	Desi		India	-	7	9	-	-	-	-	3	6	6.25	S
IC485974	ICC2484	Desi	9/1/1973	Iran	-	-	6	-	7	-	-	-	6	6.33	S
ICC6657		-	10/6/1974	Iran	-	8	7	8.5	-	-	-	3	6	6.5	S
IC269305	P626-1	Desi	9/1/1973	India	-	5	-	-	-	-	-	8	-	6.5	S
IC209375	ICC4887	Desi	30/11/1997	India	-	5	-	-	-	-	-	8	-	6.5	S
EC267154	FLIP87-505C	Desi	19/10/1988	Syria	5	-	-	-	6	-	9	-	-	6.67	S
ICC2757		-	9/1/1973	Iran	-	-	6	8	-	-	-	-	6	6.67	S
EC528345B	ICC7600	Desi	5/8/1974	Italy	5	-	-	-	-	-	9	-	-	7	S
IC209355	ICC4753	Desi	30/11/1997	India	-	5	-	-	-	-	-	9	-	7	S
IC244350	ICC1881	Desi	9/1/1973	India	-	6	-	-	-	-	-	8	-	7	S
ICC4330		Kabuli	9/1/1973	Iran	-	-	3	-	-	-	-	-	2	2.5	R
ICC3625		Kabuli	6/10/1974	Iran	-	-	2	-	-	-	-	4	-	3	R
ICC3687	EC482202	Desi	9/1/1973	Iran	-	-	4	-	-	-	-	-	2	3	R
ICC7080	PI360554	Desi	6/10/1974	Iran	-	-	5	-	-	-	-	2	-	3.5	MR
ICL3733		Desi	6/6/1999	Syria	-	-	3	-	-	-	-	-	4	3.5	MR
ICC3775	P-43931	Desi	9/1/1973	Iran	-	-	5	-	-	-	-	-	2	3.5	MR
IC299231	ICC13997	Desi	7/5/1985	Ethiopia	-	-	4	-	-	-	-	-	4	4	MR
EC482508	ICC4213	Kabuli	9/1/1973	Iran	-	-	3	-	-	-	-	-	6	4.5	MR
ICC4407	P-5392	Desi	9/1/1973	Iran	-	-	3	-	-	-	-	-	6	4.5	MR
ICC3596	P-4266	Desi	9/1/1973	Iran	-	-	5	-	-	-	-	-	4	4.5	MR
ICC4253		Desi	9/1/1973	Iran	-	-	5	-	-	-	-	-	4	4.5	MR
ICC4295	P-5245	Kabuli	9/1/1973	Iran	-	-	5	-	-	-	-	-	4	4.5	MR
IC35047		Desi		India	-	-	5	-	-	-	-	-	4	4.5	MR
EC441779	ICL3600	Desi	6/6/1999	Syria	-	-	4	-	-	-	-	-	6	5	MR
ICC4260	P-5201	Desi	9/1/1973	Iran	-	-	4	-	-	-	-	-	6	5	MR
ICC4321	P-5282	Desi	9/1/1973	Iran	-	-	4	-	-	-	-	-	6	5	MR
EC555200	ICC12792	Desi	12/1/2005	Ethiopia	-	-	5	5.5	7	-	-	4	-	5.38	S
EC441959	ICL4381	Desi	6/6/1999	Syria	-	-	2	-	-	-	-	-	9	5.5	S
ICC3230	P-37882	Desi	9/1/1973	Iran	-	-	5	-	-	-	-	-	6	5.5	S
ICC4301	P-5253	Desi	9/1/1973	Iran	-	-	5	-	-	-	-	-	6	5.5	S
ICC4304	P-52541	Desi	9/1/1973	Iran	-	-	5	-	-	-	-	-	6	5.5	S
IC396753		Desi		India	-	-	-	8.5	-	-	-	-	5	6.75	S
IC267112		Kabuli		India	-	7	-	6	-	-	-	8	-	7	S

- = respective accession was not evaluated in the season/location.

### *Ascochyta rabiei* inoculum preparation

At PAU, Ludhiana, isolate 8 of race 6(3968) was used for mass multiplication, whereas, at HAREC, HPKV, Dhaulakuan, local prevalent isolates were used for creating artificial epidemics [24]. As per the recent studies on revealing pathogenic variability and diversity existing in *A*. *rabiei*, the local prevalent isolate at Dhaulakuan are AR5, AR6, AR7 [25, 26]. The inoculums was mass-multiplied on Kabuli chickpea seed media. For preparing inoculums, seeds of Kabuli chickpea were soaked overnight in water and about 50 g of the soaked seeds were transferred in 250 ml flasks. These were sterilized by autoclaving twice at 121°C (15 psi) for 25 min. Sporulated inoculum of the *A*. *rabiei* isolate grown on dextrose agar was transferred aseptically onto the seeds in the flask. The inoculated flasks were incubated at 20 ± 0.5°C with a 12 h alternate light and dark period. The flasks were frequently shaken to avoid clumping of inoculum. Abundant conidial production was obtained by harvesting in water after 6–8 days.

### Seed planting, artificial pathogen inoculation and disease assessment

The planting was done during first week of November in each winter season. Each accession was grown in 3 m row length spaced at 40 cm and replicated twice at all the locations. Indicator-cum-infector rows of susceptible checks L550 and JG62 were planted after every five test rows. To keep at least minimum threshold level of the disease and uniform spread throughout the experimental field, artificial inoculation of the pathogen was taken to avoid any chance of scape of the disease. Inoculation was done at the pre-flowering stage *i*.*e*. by January last week. The field was irrigated in the morning hours on the day of inoculation. The inoculation was done in the evening by spraying spore suspension 4 × 10^4^ spores ml^-1^. The relative humidity of above 85% was maintained by running perfo-spray system during the day time from 10.00 to 16.00 h for 21 days. The level of disease severity was recorded on 0–9 scale, modified from the method given by Jan and Wiese, 1991 [27], where 0.0–1.0 = no visible disease symptom on any plant; 1.1–3.0 = disease lesions visible on less than 10% of the plants, no stem girdling; 3.1–5.0 = lesions visible on up to 25% of the plants, stem girdling on less than 10% plants but little damage; 5.1–7.0 = lesions present on most of the plants, stem girdling on 50% of plants; 7.1–9.0 = lesions coalesced on plants, stem girdling present in more than 50% of plants. Based on the disease severity score, accessions were categorised for their reaction to AB infection as follows: 0.0–1.0 = asymptomatic or highly resistant (HR); 1.1–3.0 = resistant (R); 3.1–5.0 = moderately resistant (MR); 5.1–7.0 = susceptible (S); and 7.1–9.0 = highly susceptible (HS). The disease symptoms started appearing after 10–15 days of inoculation and disease scoring was done when the susceptible checks showed the disease severity score of 9.0.

### Statistical analysis

The Wilcoxon Signed Rank test, a non-parametric test was done using IBM SPSS Statistics software. The test was done for paired samples with α = 0.05 to test the H_0_ hypothesis. The H_0_ hypothesis of the test was that there was no difference in AB disease severity level between two experimental locations. A common set of accessions (1,230), which were screened in three seasons i.e. rabi 2014–15, 2015–16 and 2016–17 were used to test the hypothesis. The appropriate corrections was applied to overcome the impact of ties and continuity in the data set. The asymptotic p-value was computed using exact method.

## Results

**Fig 3:**
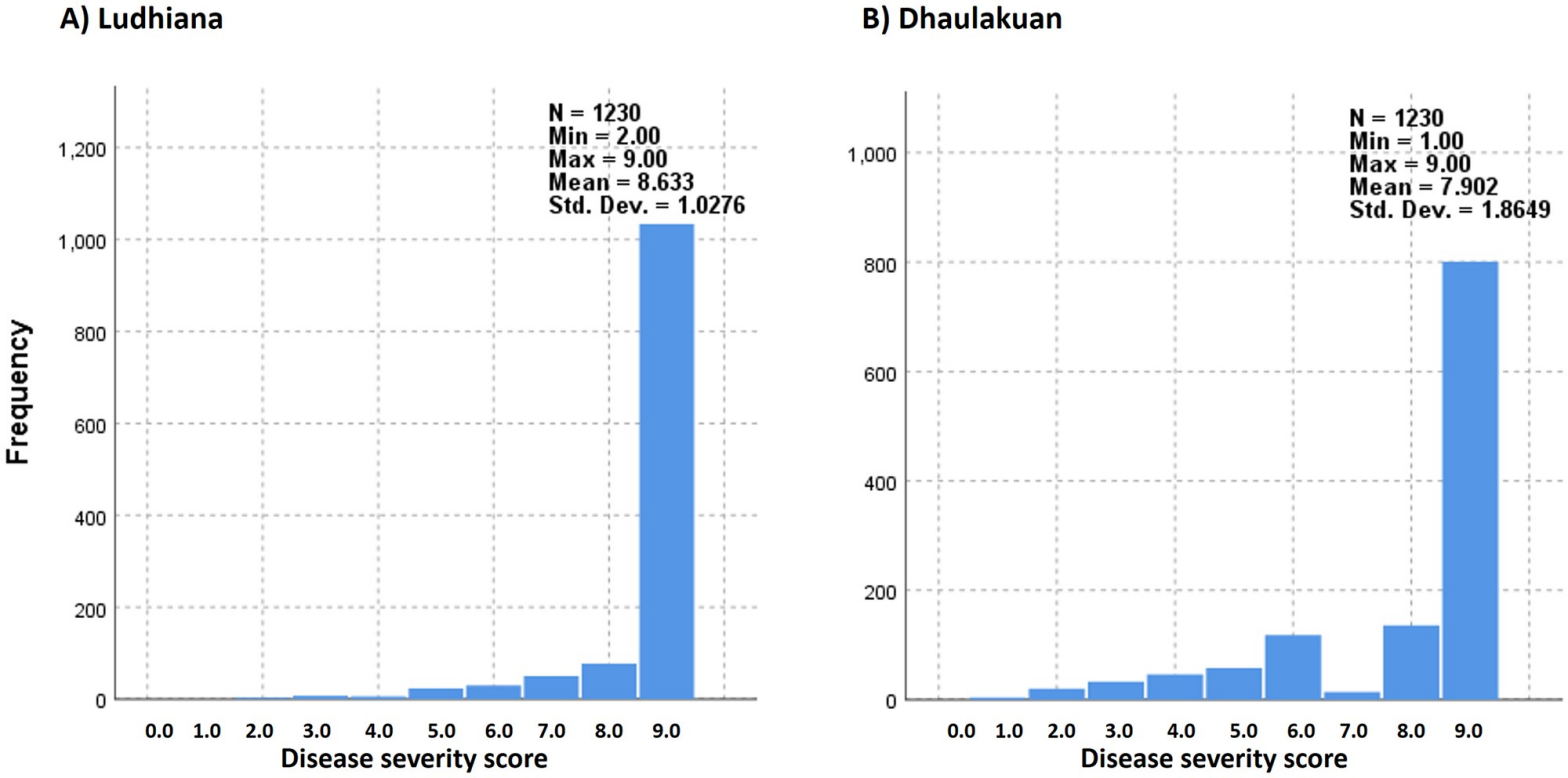
Classification of chickpea germplasm based on their response to ascochyta blight disease. In this graphical representation 1,230 accessions are included from screening experiment of three seasons (2014–15 to 2016–17) at Ludhiana (A) and Dhaulakuan (B) locations.

The main objective of this study was to identify new sources of resistance against the *A*. *rabiei* disease in Indian National Genebank chickpea collections. Therefore, wide range of cultivated chickpea genetic diversity was explored against the disease and it was found that chickpea accessions with robust resistance are very rare. A total of 1,970 chickpea accessions comprised of Indigenous accessions (1,567) and exotic accessions (403 representing 20 countries were screened against this disease (Table 1). The disease severity was very high in both the locations and only very few chickpea accessions having resistance against the disease were identified (Table 2 and Fig 3). Based on the mean value of disease severity score (0–9) across the locations and seasons, only 0.5% of total accessions were scored as resistant, followed by moderately resistant (1.6%), susceptible (10.6%) and rest were highly susceptible (87.4%). When resistant and moderately resistant types of accessions were traced back to their origin country, it was observed that frequency of such accessions was comparatively higher in accessions originated from Southwest Asian countries particularly Iran and Syria than the accessions originated from Indian sub-continent. Total 338 accessions originated from Southwest Asia. Out of which five accessions (1.5%) were found resistant, 22 moderately resistant (6.5%), 45 susceptible (13.3%) and 266 highly susceptible (78.7%). Whereas, only four resistant (0.26%), nine moderately resistant (0.57%) and 161 susceptible (10.27%) were found out a comparatively large population (1,567 accessions) originated from India (Table 1).

Screening across both the locations indicated that the chickpea germplasm is highly susceptible for the disease and there was no highly resistant/immune type of accession against the pathogen. However, some promising accessions in the category of resistant (score 1–3) and moderately resistant (score 3.1–5) were identified and also validated in multiple seasons (Table 2). During winter 2014–15, at Ludhiana centre four accession i.e. EC220109, EC267154, EC267240 and EC528345B showed moderately resistant reaction with a score of 5.0. During the same season at Dhaulakuan location, 40 accessions were found with disease severity score less than 3.0 of which 12 accessions i.e. IC83811, EC267265, EC267272, EC267293, EC489910, EC554996, IC83389, IC83390, IC83453, IC83774, EC220109 and EC267240 were found resistant (severity score ≤ 2). Screening during winter 2015–16 at Ludhiana centre resulted in identification of eight accessions i.e. IC114477, IC275447, IC209641, IC209317, IC209355, IC209375, IC244185 and IC269305 with their respective score of 2, 3, 4 and rest with 5 (Table 2). IC275447 was found resistant for four seasons i.e. winter 2015–16, 2017–18, 2018–19, 2019–20 and two locations i.e. PAU Ludhiana and Dhaulakuan. The accession IC114477 was also found resistant (score ≤ 2) in three seasons i.e. winter 2015–16, 2017–18, 2018–19 and at both locations (Table 1). A Kabuli grain type accession EC267301 was found resistant at Dhaulakuan in winter 2015–16 which was further validated and found resistant at Ludhiana location also during 2016–17, 2018–19 and 2019–20. Accessions EC220109 was found resistant during 2014–15 in both the locations and it was further validated as resistant (severity score 3) during 2018–19. During 2019–20, seven new accessions viz. IC297322, IC275501, IC275448, IC244328, IC41651, IC267114, ICC4061 with moderate level of resistance were identified. There are few other promising accessions which are still being further validated in coming seasons. However, we could narrow down five promising accessions in the category of resistant (score 1–3) which were found constantly resistant across the locations and in multiple seasons. These accessions are IC275447, EC267301, IC117744, IC248147 and EC220109. (Table 2 and Fig 2). Promising accessions which were observed resistant or moderately resistant are highlighted in S2 Data.

It was observed that the disease severity over the locations was not consistent with respect to individual accessions. Graphical representation of germplasm based on their severity scale indicated highly skewed distribution. Most of the accessions belong to highly susceptible category, followed by susceptible type and it was also observed that the disease severity at Ludhiana location was comparatively higher than the Dhaulakuan location (Fig 3).

The experimental sites i.e. Ludhiana and Dhaulakuan are hotspot region to the pathogen, but the screening results shows that the severity of the disease was different. It has been observed that the severity of the disease was relatively higher at the Ludhiana compared to Dhaulakuan. Statistical analysis was done to understand whether the results obtained from both the locations are significantly different from each other or not in terms of their disease severity. The Wilcoxon Signed Rank test on paired set of accessions (1,230) indicated that the distribution for the disease severity was not the same (Table 3). The null hypothesis (H_0_) was rejected and alternate hypothesis (Ha) i.e. ‘median rank of the two dependent samples is not the same’ was accepted. Therefore, it was speculated that the variation in disease severity between two locations may be due to two reasons i.e. varied environmental conditions or pathogen racial differences. However, results obtained from the disease screening for paired set of accessions also indicate that the disease severity varies for accessions between locations, i.e. accessions showing resistance in one location may be susceptible in another locations and vice versa. Therefore, it was established that the variation in disease severity might be due to racial difference between two locations.

**Table 3:** Summary table of Wilcoxon Signed Rank test on paired set of accessions for Dhaulakuan and Ludhiana disease screening locations.

		N	Mean Rank	Sum of Ranks
Dhaulakuan—Ludhiana	Negative Ranks	337	220.45	74290.00
	Positive Ranks	72	132.71	9555.00
	Ties	821		
	Total	1230		
Standard Deviation: 2374.02				
Standardized Test Statistic: -13.63				
Asymptotic Sig.(2-sided test): 0.00				

## Discussion

The Ascochyta blight disease has become a major limiting factor for yield enhancement in chickpea in all chickpea growing areas. *Ascochyta rabiei* is the causal organism for the blight, which is known to be highly variable fungus [5, 26]. The heterothallic nature of the fungus and sexual mating between two mating types i.e. MAT-1 and MAT-2 ensures sexual recombination and new allelic combination [21]. The rapid evolving genome of the fungus outpace the existing genic/allelic combinations responsible for defence mechanism in host plant. Therefore, the rapid breakdown of resistance in earlier identified donors as well as in varieties are reported [5, 19–21]. Understanding the host resistance mechanism against the disease is the perquisite for breeding resistant varieties. However, numerous studies done to understand the inheritance of the disease resistance indicated quite a few types of inheritance, which further complicate breeding resistance. There were some studies which showed that the host resistance is governed by single dominant gene [28, 29], two dominant complementary genes [30]. A study based on two interspecific RIL populations shows that the three recessive and complementary major genes with several modifiers are responsible for the resistance [31]. They further mapped the major QTLs (QTL-1 and QTL-2) in linkage group 6 and 1[30]. There are several other studies indicating that the loci governing the disease resistance are quantitative in nature [32–36]. A study showed that the inheritance is genotype specific [37]. Out of six resistant genotypes used in crossing, five showed independent single dominant gene action while one genotype showed single resistant gene action. Further a study on fifteen resistant genotypes in combination of screening against four races indicated that host resistance is genotype specific [11]. Therefore, it is imperative to find new sources of the disease resistance which may have novel defence mechanisms. The new sources will play the crucial role in developing genotypes with long-lasting resistance amid fast evolving pathogen. Therefore, this study was undertaken with the objective to identify new sources of resistance against the *A*. *rabiei* in Indian chickpea collections which is poorly explored. Both the experimental locations are designated hotspots for the disease where frequent severe disease incidences are observed under natural field conditions. However, artificial optimal conditions given for disease development were helpful in uniform spread of the disease in field and to avoid any escape. The screening and validation results indicate that the chickpea accessions with robust resistance are very rare in cultivated chickpea gene pool. However, we have identified five accessions *viz*. IC275447, IC117744, EC267301, IC248147 and EC220109 displaying constant resistance against the disease across the locations in multiple seasons (Table 2 & Fig 2). From the literature survey we could also find out that the promising accessions identified in this study have not been reported earlier thus making them as novel and valuable genetic resources which can be used as donors for the chickpea improvement programs. However, the resistant germplasm lines may be further tested in other epidemic locations of the *A*. *rabiei* representing the other races in order to identify lines having broader resistance.

It was observed that disease reaction of an accession varies from one screening location to another. Therefore, it appears that accessions such as IC275447, IC117744, EC267301, IC248147 and EC220109 which were found resistant in both the locations have broader resistance, which might be governed by multiple factors/genes. Similar differential pathogenicity results were reported in an extensive chickpea screening done against the disease from 1978 to 1982 in more than 11 countries [38]. This indicates the presence of variability in endemic pathogen races of the experimental locations. Comparatively higher frequency of R and MR type accessions originated from Southwest Asian region might be due to the variation in pathogen pressure between these regions. Chickpea cultivation in some areas are more affected by frequent epidemics of the disease due to more favourable weather conditions (cool, wet and cloudy) for pathogen spread during the growing season. The more stringent natural selection pressure of the pathogen over the years might have resulted in enhanced frequency of resistant alleles/QTLs in the chickpea germplasm from this region. The ascochyta blight is one of the major constraint of chickpea in WANA (Western Asia and North Africa) and Southern Europe [10, 17, 39]. In India the disease is a major problem of northern states particularly Punjab, Uttarakhand and parts of Himachal Pradesh [40]. These are also the regions where the major chunk of the chickpea diversity evolved. Therefore, we speculate that the chickpea germplasm originated from this region may be better source of identifying novel sources of the pathogen resistance.

Though it has been a period of a century since the disease was first appeared and has spread across the continents, very limited success has been achieved in identifying chickpea genotypes with robust and broader resistance. Several small scale chickpea germplasm screenings have been reported [41–43] which resulted in identification of resistant or tolerant chickpea accessions. In a study three chickpea accessions i.e. PI 559361, PI 559363 and W6 22589 were identified as resistant from total 44 accessions screened [19]. But another report showed that the resistant sources are rare [44]. In a study 29 resistant lines were reported [45]. These were identified from 150 elite chickpea breeding lines which were screened using combination of methods i.e. cut-twig based lab screening method and commonly used field screening method. Screening of 19,343 global chickpea germplasm collection (12,749 desi and 6,594 Kabuli types) against six races of *A*. *rabiei* at Tel Hadya, Syria, between 1979 and 1991 could only result in finding three desi type (ICC4475, ICC6328, and ICC12004) and two Kabuli type (ILC200 and ILC6482) chickpea accessions resistant against all six races. This work was a joint venture of International Crops Research Institute for the Semi-Arid Tropics (ICRISAT), Patancheru, India and International Center for Agricultural Research in the Dry Areas (ICARDA), Syria. The identified resistant chickpea lines are the only main source used in current national and international breeding programs.

Recent past experiences of *A*. *rabiei* epidemics and large scale germplasm evaluation indicated that the disease resistance sources in chickpea genepool are rare and finding robust sources of resistance against all the prevalent isolates of the pathogen would be the best sustainable option. As a result of climate change increased frequency of non-seasonal erratic winter rains and medium to high speed winds are becoming more common and the trend may remain the same in near future, which are likely to create more congenial environment for the disease spread. Therefore, identifying promising resistant sources within cultivated chickpea primary genepool will be of strategic importance. Further, identification of genes/QTLs and pyramiding them in elite chickpea cultivars will be the most economical and sustainable way forward. The resistant germplasm identified in this study will have direct utilization to combat the problem to sustain chickpea production.

## Supporting information

S1 Data Passport data for chickpea germplasm. (XLSX)

S2 Data Disease scoring data for each location and season. (XLSX)
